# Intelligence across humans and machines: a joint perspective

**DOI:** 10.3389/fpsyg.2023.1209761

**Published:** 2023-08-17

**Authors:** Tiago Buatim Nion Da Silveira, Heitor Silvério Lopes

**Affiliations:** ^1^Computational Intelligence Laboratory (LABIC), Federal University of Technology – Paraná, Curitiba, Brazil; ^2^Polytechnic School, University of Vale do Itajaí, Itajaí, Brazil

**Keywords:** human intelligence, computational intelligence, artificial intelligence, intelligence measurement, IQ, ChatGPT, CHC model, semantics

## Abstract

This paper aims to address the divergences and contradictions in the definition of intelligence across different areas of knowledge, particularly in computational intelligence and psychology, where the concept is of significant interest. Despite the differences in motivation and approach, both fields have contributed to the rise of cognitive science. However, the lack of a standardized definition, empirical evidence, or measurement strategy for intelligence is a hindrance to cross-fertilization between these areas, particularly for semantic-based applications. This paper seeks to equalize the definitions of intelligence from the perspectives of computational intelligence and psychology, and offer an overview of the methods used to measure intelligence. We argue that there is no consensus for intelligence, and the term is interchangeably used with similar, opposed, or even contradictory definitions in many fields. This paper concludes with a summary of its central considerations and contributions, where we state intelligence is an agent's ability to process external and internal information to find an optimum adaptation (decision-making) to the environment according to its ontology and then decode this information as an output action.

## 1. Introduction

On the one hand, divergences and contradictions in the definition of concepts between areas of knowledge can be ignored or minimized when such areas have little or no relationship (e.g., structure in civil engineering carries a different meaning of structure in psychology). On the other hand, we must be rather careful when a given concept is used in areas of distinct epistemic bases but with very related applications. This is the case with intelligence. From the etymological point of view, the term intelligence originates from the Latin language and designates the ability to understand. Over the years, the term has been used for the most diverse areas, from military strategies to business. Today, its lay use has been most associated with logic, good grades, and problem-solving abilities (Cohen et al., 2009)—from a cognitive perspective.

From the beginning of the twenty-first century, the popularization of neural networks, machine learning, and deep learning applications turned the term intelligence strongly associated with computational intelligence (CI), leveraged by an increasingly human-like capacity—especially those related to semantics such as computer vision (CV) and natural language processing (NLP). Recently, the excitement caused by generative models such as Dall-E and ChatGPT for image and text generation, respectively, has contributed to reinforcing the idea of a possible human-like general intelligence with comparable semantic comprehension abilities.

Regarding psychology and computational intelligence, we should observe that the motivation in their studies on intelligence differs: psychology is marked by an interest in individual differences, which began in the 19th century with Francis Galton, while computational intelligence started with artificial intelligence in the mid-20th century, with the expectation of emulating human intelligence focusing its commonality. Despite these differences, both disciplines have contributed significantly to the rise and enhancement of cognitive science (Anderson, 2009; Russel and Norvig, 2009).

Before discussing the validity of a “general” intelligence, it is of fundamental importance to primarily discuss the generalization of the concept of intelligence, since many of them lack formalization, empirical evidence, or a measurement strategy. With this research, and assuming that intelligence is a quality or ability of a given agent or system (whether it is human, biological, or artificial), we seek to equalize such definitions of intelligence from the perspectives of computational intelligence and psychology, thus contributing to the cross-fertilization between these areas, especially for semantic-based applications.

This paper is organized as follows: the main theories and definitions of intelligence are compiled in Section 2, while Section 3 offers an overview of the methods used to measure intelligence in both computing and psychology, including the evaluation of intelligence in the relationship between humans and machines. In Section 4, we claim there is no consensus for intelligence, meaning that the term intelligence is interchangeably used in many fields with similar, opposed, or even contradictory definitions. In Section 5, we finalize this paper with this article's central considerations and contributions.

## 2. Multiple definitions of intelligence

Intelligence has historically been associated with formalism (Flynn, 2007), resulting from a positivist perspective that originated, among other things, computing itself. Computer science is formalist by definition (Sipser, 2012), although disciplines such as computational intelligence are mostly based on the information theory (Shannon, 1948). Psychology, on the other hand, started from a solid positivist influence and began considering different epistemic bases in its studies since the emergence of psychodynamics (Solms and Turnbull, 2010). In this section, we will review the main concepts and definitions of intelligence found in computational intelligence (Section 2.1) and psychology (Section 2.2) that support or oppose each other.

### 2.1. Definitions from computational intelligence

As defined by Russel and Norvig (2009), an intelligent agent is an entity that perceives and acts in a given environment through sensors and actuators, respectively. In this definition, the agent function, particular to each agent, determines the action to be taken by the agent in response to any perceived stimulus and is implemented through a program—the latter running on a hardware architecture containing sensors and actuators. The authors point out countless ways to implement the same agent function so that the programs present differences in efficiency, compactness, and flexibility. Therefore, the appropriate program design that will implement the agent function will depend on the nature of the environment in which this agent will be inserted. In the authors' view, this process defines artificial intelligence (AI), which they establish as the science of designing agents.

**Figure 1:**
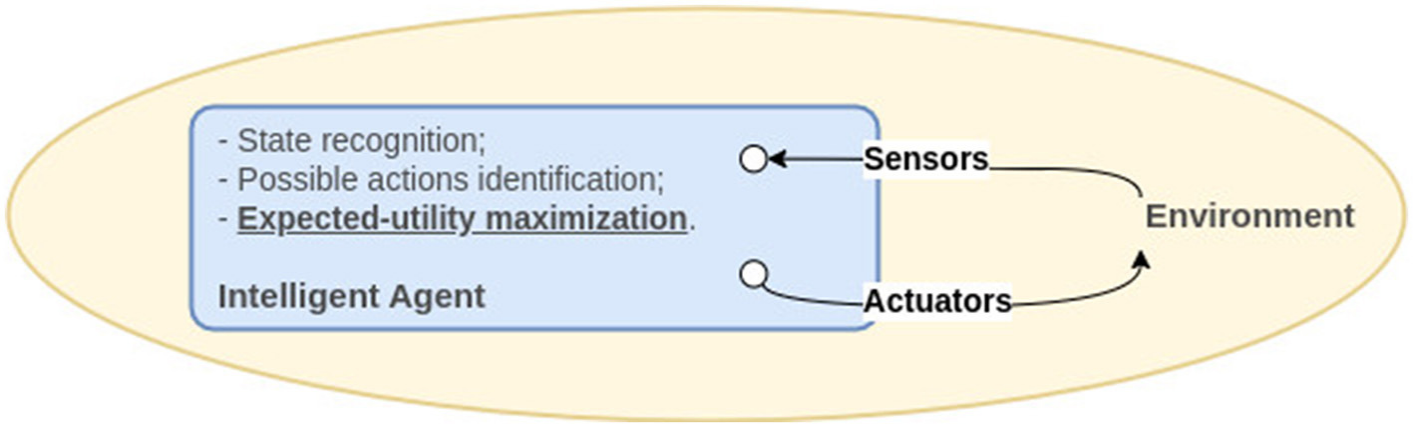
Diagram of a model-based, utility-based agent. An intelligent agent perceives its environment through sensors and acts in this environment through actuators in a way to maximize its utility. Adapted from Russel and Norvig (2009).

The same authors suggest that intelligent agents can be categorized into (i) simple reflex agents, when they respond directly to perceptions; (ii) model-based reflex agents, when the response to perceptions occurs through internal states that are not evident; (iii) goal-based agents, whose actions are directed to achieve a previously-defined objective; and, finally, (iv) utility-based agents, who try to maximize their expected utility. In any case, the agent reads the environment state through sensors and acts on the environment through actuators, as shown in Figure 1. In this conception, agents improve themselves through learning, usually based on penalties and rewards. Interestingly, the categorization of intelligent agents into reflex, goal-based, and utility-based types echoes the concept of behaviorism in psychology, where behavior is understood as a response to stimuli in the environment, directed toward a goal or outcome, and shaped by rewards and punishments.

From the Russel and Norvig's conception, a human would be defined as a utility-based agent: from an internal model of the world (i.e., the environment), the agent would choose the action that would maximize the expected utility, computed through the average of all possible output states and weighted by the probability of each output. The authors consider happiness as the agent function, which means a human agent would operate to become happier. It should be noticed that the attribution of happiness to the expected utility corroborates the ideas from behaviorism (Skinner, 1965), but does not correspond to the subjective conceptions of happiness or pleasure that can be found in Freud (1920) and Siegel (2010) nor by the concept of expected utility in situations of uncertainty addressed by Gilboa (2010).

**Figure 2:**
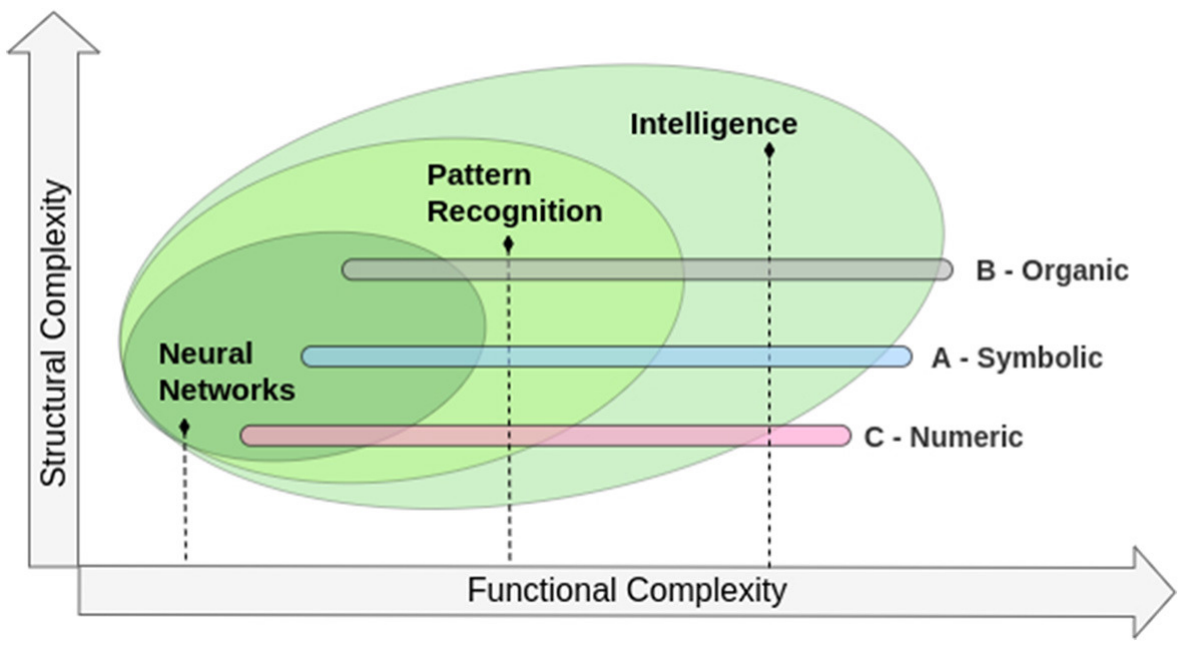
Relationship between biological (B), artificial (A), and computational (C) organizations of neural networks (NN), pattern recognition (PR), and intelligence (I). Adapted from Bezdek (2016).

Bezdek (1992) proposed the distinction between artificial, biological, and computational intelligence—what he called the “ABCs of neural networks (NN), pattern recognition (PR), and intelligence (I),” whose relationship between concepts is represented through its acronyms in Figure 2. In such categorization, CI is a subset of AI which, in turn, is a subset of biological intelligence in terms of complexity. The same relationship is established for NN: a subset of PR, and, again, in terms of complexity, a subset of intelligence. In a more recent publication on the subject, Bezdek (2016) recognizes that, although this distinction remains valid, the borders between AI and CI are increasingly blurred.

For instance, deep neural networks, which are a cornerstone of artificial intelligence, are inspired by the structure and function of the human brain, blurring the line between artificial and biological intelligence. Similarly, computational intelligence techniques such as fuzzy logic and genetic algorithms have found applications in a wide range of fields, from finance to medicine, demonstrating the versatility and power of these approaches. Therefore, while the distinction between artificial, biological, and computational intelligence remains a useful conceptual framework, it is important to recognize that the boundaries between these domains are constantly evolving and shifting.

Although without explicitly defining intelligence, Shneiderman (2020) proposes an alternative categorization for artificial intelligence based on goals. The author distinguishes AI research into two major groups: (i) those seeking to emulate human intelligence—for which the author includes “aspiration for humanoid robots, natural language and image understanding, commonsense reasoning, and artificial general intelligence”—and (ii) those seeking to build valuable applications, i.e., “AI-guided products and services” such as “instruments, apps, orthotics, prosthetics, utensils, or implements.”

Pandl et al. (2020) bring an implicit definition of intelligence by stating that “AI enables computers to execute tasks that are easy for people to perform but difficult to describe formally.” To this end, the authors segment artificial intelligence into (i) artificial general intelligence (AGI), an open-domain approach defined by Goertzel and Monroe (2017) as the human-like design of self-organizing and complex adaptive systems; and (ii) narrow artificial intelligence, a domain-specific approach in which the authors include knowledge bases and machine learning methods.

More recently, a conceptualization of intelligence based on its measurement was suggested by Chollet (2019, p. 27), who stated that “intelligence of a system is a measure of its skill-acquisition efficiency over a scope of tasks, with respect to priors, experience, and generalization difficulty.” Such a definition is quite similar to the instrumentalist attempt from psychology to define intelligence, as we will describe in the next Section.

### 2.2. Definitions from psychology

Psychology approaches intelligence as a construct—a psychometric concept designed to explain or understand a psychological phenomenon (i.e., the latent trait) from its indirect manifestations (i.e., the overt behavior). Cohen et al. (2009) bring the concept of interactionism to explain those theories that attribute intelligence to the interaction between people, consisting of biological and social factors, and these with the environment. In addition, Cohen et al. group factor-analytical theories as those concerned with identifying which factors or set of skills express intelligence. Finally, they call information processing theories those that study the mental processes that result in intelligence.

Historically, intelligence assessment has played an important and controversial role in psychology. Some authors (Simonton, 2003; Cohen et al., 2009) attribute to Francis Galton the beginning of the scientific study of individual differences, who established mathematical techniques such as correlation to measure it—a method that later on would be improved by Pearson and Spearman.

In 1904, Spearman (1961, p. 260) observed that the “correlations calculated between the measurements of different abilities (scores for tests, marks for school subjects, or estimates made on general impression)” may follow a specific mathematical relationship he called “tetrad equations”. Such equations led him to formulate what is known today as factor analysis: a statistical technique that aims to uncover the underlying factors that explain the interrelationships among a set of observed variables. The tetrad equations reflect that the observed variables (e.g., test scores, questionnaire responses) can be modeled as a combination of common and unique factors. Spearman thus formulated the “two-factor theory” of intelligence, which included the specific factor (*s*), based on the unique ones, and the general factor of intelligence, or *g* factor, which would be common to any intelligent ability and thus able to measure it.

Arthur Jensen delved into the *g* factor theory, thus defining intelligence operationally as “the first principal component of an indefinitely large number of highly diverse mental tasks” (Jensen, 1978, p. 112).

In 1905, Alfred Binet and Theodore Simon published a measuring scale of intelligence applied to Parisian children—the first to use a standardized intelligence quotient (IQ) scale. Instead of defining intelligence, Binet limited himself to describing its components: “reasoning, judgment, memory, and abstraction” (Cohen et al., 2009, p. 292). For him, intelligent behavior was a joint result of these abilities, countering Galton's idea that each could be assessed separately.

The indiscriminate and uncritical use of psychological tests in the early twentieth century led Boring (1961) to state, in 1923, that “measurable intelligence is simply what the tests of intelligence test,” thus claiming for a better definition. Flynn (2007) has called this perspective instrumentalism, defined by him as the attempt “to measure by referring to the readings of the measuring instrument,” which is thus “subject to devastating critique.”

In 1939, David Wechsler designed an intelligence test that could be applied to adults, defining intelligence as “the aggregate or global capacity of the individual to act purposefully, to think rationally, and to deal effectively with his environment” (Wechsler, 1958). His work originated the gold-standard tests for intelligence known as Wechsler Adult Intelligence Scale (WAIS) and Wechsler Intelligence Scale for Children (WISC). Wechsler's definition stands out since it does not restrict intelligence to cognitive and executive functions but, also, considers the role of conative functions, i.e., those related to affection (Cohen et al., 2009, p. 52).

In 1940s, Cattell (1941) started a work, which was later developed by Horn (1965), that led to a model where two skills were defined: crystallized intelligence (Gc), which includes acquired vocabulary and knowledge, and fluid intelligence (Gf), which is non-verbal and has few cultural influences, such as the numerical memory (Carroll, 2003). Other factors were later added by Horn: visual processing (Gv), auditory processing (Ga), quantitative processing (Gq), speed of processing (Gs), facility with reading and writing (Grw), short-term memory (Gsm), and long-term storage and retrieval (Glr) (Cohen et al., 2009, p. 297).

In the 1950s, Jean Piaget elaborated his theory of intelligence development in children (Piaget, 2003). For him, learning would occur through assimilation and accommodation processes. The first consists of absorbing new data to fit it into already-known information. The second consists of changing the registered information to fit the new one. Piaget's theory states that both processes would occur along the four development stages: (i) the sensorimotor stage, until 2 years, with abilities of goal-directed and intentional behavior, coordination and integration of the five senses, and recognition of the world and its objects as permanent entities; (ii) the preoperational stage, from 2 to 6 years, with concepts understanding largely based on vision, contextual comprehension typically based on a single or obvious aspect of the stimulus, irreversible thought (focus on static states of reality, without making relations between them), animistic thinking (attributing human qualities to non-human objects); (iii) the concrete operations stage, from 7 to 12 years, with conservation of thought (world's attributes remain stable), part-whole problems and serial ordering tasks solving, reasoning based on direct experience, problem-solving through more than one aspect, and present and historical differentiation; and (iv) formal operations, over 12 years, with reasoning also based on indirect experience, hypotheses and test formulations (systematic thinking), complex reasoning through several variables (systematic perception), evaluation of the own thought, and deductive reasoning.

Piaget's theory of cognitive development has been influential in shaping the understanding of human intelligence and its development. In the context of computational intelligence, these concepts are relevant as they provide insights into the development of intelligent systems. Piaget's theory emphasizes the importance of assimilation and accommodation processes in learning, which can be linked to machine learning algorithms that use previously learned information to make predictions or decisions based on new data. In the early stages of development, AI systems may rely on simple rule-based approaches, similar to Piaget's sensorimotor stage. As the system develops, it can progress to more complex approaches that involve reasoning and problem-solving, similar to Piaget's concrete and formal operational stages. It is worth noticing that, according to Piaget's model, only in the last stage, the formal operations period, would a child be able to abstract and fully express formal and semantic abilities. Such skills are not yet fully performed by computational intelligence due to the lack of representation structures for such tasks. In this way, representation learning is a current research topic in CI (Goodfellow et al., 2016) and interfaces the findings from psychology as in Palmer (1999) and Sterling and Laughlin (2015).

The information processing paradigm, described by Palmer (1999, p. 70) as “a way of theorizing about the nature of the human mind as a computational process”, started to be employed in psychological theories from the middle of the twentieth century. Alexander Luria, considered the precursor of neuropsychology (Cohen et al., 2009, p. 300), was the first to conceptualize intelligence from this approach. Luria has demonstrated two ways of information processing: (i) simultaneous or parallel and (ii) successive or sequential. The first would be associated with semantic attribution tasks, while the second would involve tasks that demand attention and linear execution, such as spelling a word. Such a paradigm provides a framework for understanding how intelligent systems process information. The simultaneous or parallel processing, which is associated with semantic attribution tasks, can be linked to approaches such as deep learning in computational intelligence, where multiple layers of neurons process information simultaneously to extract features of different abstraction levels. The successive or sequential processing, associated with tasks that demand attention and linear execution, can be linked to approaches such as reinforcement learning, where an agent learns by interacting with an environment in a sequential manner.

Gottfredson (1997) defined intelligence as “a highly general information processing capacity that facilitates reasoning, problem-solving, decision-making, and other higher order thinking skills.” Also situated in the information processing paradigm, Sternberg (2003) proposed the triarchic theory of intelligence, composed of analytical, creative, and practical aspects. Later, Sternberg (2019) proposed the theory of adaptive intelligence, questioning the general factor of intelligence, its metrics, and theoretical assumptions. His thesis is related to the environmental impact caused by humanity and questions how intelligent such actions are. Thus, he promotes a debate between such conceptions of intelligence from a biological and optimal perspective.

The consideration of a general intelligence factor, the *g factor*, started to be used again after Carroll's work, which divided intelligence into three strata of abilities: specific, broad, and general (Carroll, 2003). Flynn (2007, p. 48) argued against the *g* factor, stating it does not provide a robust definition of intelligence but limits it to a comparison approach. He also claimed that intelligence's social and physiological aspects are reduced to the possibility of enhancing or not the significance of *g*. As an alternative, Flynn proposes a theory that integrates physiological, individual, and social aspects of intelligence, which he calls the BIDS model—an acronym indicating the brain; the individual differences, which he associates with the *g* factor; and the society.

Recently, McGrew (2009) unified the theories of Cattell-Horn and Carroll in what he called the CHC-Theory. The Cattell-Horn model considered a total of eight abilities distributed through crystallized (Gc) and fluid (Gf) factors of intelligence. In turn, Carroll's model proposed that intelligence should be divided into three hierarchical strata: the first, of specific skills; the second, of complex factors such as Gf and Gc; and the third, the general intelligence factor, or *g*.

**Figure 3:**
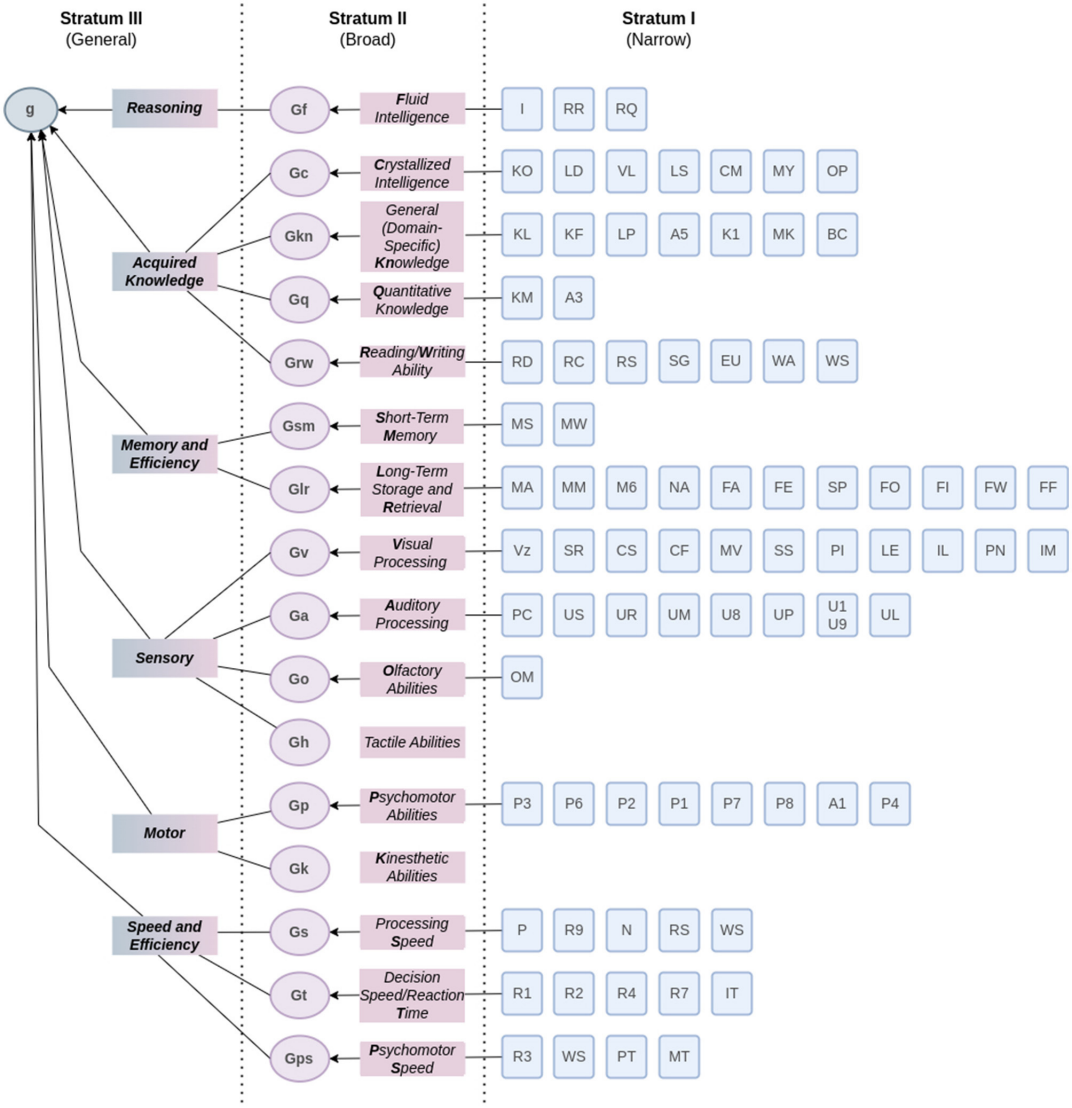
CHC theory of intelligence. Stratum I represents the narrow abilities, as defined and detailed in Schneider and McGrew (2012). Stratum II represents the broad abilities, grouped by Flanagan and Dixon (2014) in reasoning (Gf), acquired knowledge (Gc, Gkn, Gq, Grw), memory and efficiency (Gsm, Glr), sensory (Gv, Ga, Go, Gh), motor (Gp, Gk), and speed and efficiency (Gs, Gt, Gps). Stratum III represents the factor *g* of general intelligence. Adapted from Flanagan and Dixon (2014).

The CHC-model integrates them in the way schematically represented in Figure 3. Some broad abilities (stratum II) are composed of narrow abilities (stratum I) as follow: **fluid intelligence (Gf)** relates to induction (I), general sequential reasoning (RG), and quantitative reasoning (RQ); **crystallized intelligence (Gc)** relates to general (verbal) information (KO), language development (LD), lexical knowledge (VL), listening ability (LS), communication ability (CM), grammatical sensitivity (MY), and oral production and fluency (OP); **general (domain-specific) knowledge (Gkn)** relates to foreign language proficiency (KL), knowledge of signing (KF), skill in lip-reading (LP), geography achievement (AS), general science information (K1), mechanical knowledge (MK), and knowledge of behavioral content (BC); **quantitative knowledge (Gq)** relates to mathematical knowledge (KM), and mathematical achievement (A3); **reading/writing ability (Grw)** relates to reading decoding (RD), reading comprehension (RC), reading speed (RS), spelling ability (SG), English usage knowledge (EU), writing ability (WA), and writing speed (WS); **short-term memory (Gsm)** relates to memory span (MS), and working memory (MW); **long-term storage and retrieval (Glr)** relates to associative memory (MA), meaningful memory (MM), free-recall memory (M6), naming facility (NA), associational fluency (FA), expressional fluency (FE), sensitivity to problems/alternative solution fluency (SP), originality/creativity (FO), ideational fluency (FI), word fluency (FW), and figural fluency (FF); **visual processing (Gv)** relates to visualization (Vz), speeded rotation (spatial relations) (SR), closure speed (CS), flexibility of closure (CF), visual memory (MV), spatial scanning (SS), serial perceptual integration (PI), length estimation (LE), perceptual illusions (IL), perceptual alternations (PN), and imagery (IM); **auditory processing (Ga)** relates to phonetic coding (PC), speech sound discrimination (US), resistance to auditory stimulus distortion (UR), memory for sound patterns (UM), maintaining and judging rhythm (U8), absolute pitch (UP), musical discrimination and judgment (U1 U9), and sound localization (UL); **olfactory processing (Go)** relates to olfactory memory (OM); **tactile abilities (Gh)** has no narrow ability; **pscyhomotor abilities (Gp)** relates to static strength (P3), multilimb coordination (P6), finger dexterity (P2), manual dexterity (P1), arm-hand steadiness (P7), control precision (P8), aiming (A1), and gross body equilibrium (P4); **kinesthetic abilities (Gk)** has no narrow ability; **processing speed (Gs)** relates to perceptual speed (P), rate-of-test-taking (R9), number facility (N), reading speed (fluency) (RS), and writing speed (fluency) (WS); **decision speed/reaction time (Gt)** relates to simple reaction time (R1), choice reaction time (R2), semantic processing speed (R4), mental comparison (R7), and inspection time (IT); **psychomotor speed (Gps)** relates to speed of limb movement (R3), writing speed (fluency) (WS), speed of articulation (PT), and movement time (MT).

In fact, the CHC-model adds the *g* factor to Cattell-Horn's theory and seeks empirical evidence for its validation. For each broad ability (stratum II) in the CHC-model, a set of narrow abilities (stratum I) is considered. As observed by Flanagan and Dixon (2014), the CHC theory is “a dynamic model that is continuously reorganized and restructured based on current research”, a statement reaffirmed by the last update on the CHC model where some factors were removed or rearranged (Schneider and McGrew, 2012).

For last, we must consider that human intelligence allows us to handle both accurate and vague information by computing with numbers and words. This is consistent with the Bayesian brain theory (Friston, 2010; Pouget et al., 2013), one of the most accredited theories in neuroscience sustaining “the brain both represents probability distributions and performs probabilistic inference” based on sensory input and prior models. Human decision-making based on incomplete information is also well-modeled by fuzzy logic (Zadeh, 2000). Recently, a close link between Fuzzy logic and Bayesian inference has been established (Gentili, 2021), shedding light on a possible unified perspective for intelligent behavior.

## 3. Measuring intelligence

As claimed by Cohen et al. (2009, p. 303), “how one measures intelligence depends in large part on what one conceives intelligence to be.” That said, in this section, we will describe the main intelligence measures supported by the theories and definitions given in Section 2.

### 3.1. Intelligence measurement in computational intelligence

Russel and Norvig (2009) bring an economic perspective to the field. To them, a performance measure is necessary to evaluate a given agent's behavior in a given environment. Therefore, a rational agent would act to maximize its expected value as a performance measure, that is, maximize its expected utility. According to Gilboa (2010), such a proposition is only reasonable when we understand the utility's meaning and know the probability of such an event, which usually does not occur when dealing with applications under uncertainty.

Inspired by the *g* factor of intelligence and its measure, the intelligence quotient (IQ), some researchers proposed an equivalent metric for artificial intelligence: the machine intelligence quotient (MIQ). Park et al. (2001) proposed a framework for MIQ measurement in human-machine cooperative systems. These authors affirm that “machine intelligence is the ability to replicate the human mental faculty and to perform human-like.” Following it, they define MIQ as “the measure of autonomy and performance for unanticipated events.” Actually, from their definitions, both uses of intelligence relate to different constructs or phenomena.

Another approach is offered by Ozkul (2009), who defines MIQ as the difference between the control intelligence quotient (CIQ) and human intelligence quotient (HIQ). In his work, HIQ is posed as “the intelligence quantity needed from the human controller for controlling the system,” while CIQ is defined as “the total intelligence required for carrying out all the tasks in the man-machine cooperative system.” Important to notice that an independent definition of intelligence was not provided. Liu and Shi (2014), in turn, compare the performance of the Internet with that of the human brain network and propose a shallow equivalence with the human IQ without accounting for the assumptions of validity and precision necessary for psychometry.

Regarding machine learning and deep learning, the measurement of intelligence is replaced by some kind of performance evaluation. As pointed out by Goodfellow et al. (2016), the performance measure is usually task-specific and it is obtained through well-defined measurements such as accuracy, precision, and recall.

### 3.2. Intelligence measurement in psychology

If, on the one hand, a definition of intelligence is essential for computation in order to develop better systems and agents, on the other hand, for psychology, its primary justification comes from the need to assess both children and adults, to identify their abilities and limitations due to traumas, illness, special skills, and any other diagnostic requirement.

Most of the theories for intelligence discussed in Section 2.2 sustain an intelligence measuring instrument. It is not the scope of this work to detail each of them. For illustrative purposes, we will follow the discussion based on Wechsler's subtests. In this instrument, the *g* factor is measured as the full-scale IQ (FSIQ), which is calculated from the verbal IQ (VIQ) and performance IQ (PIQ). VIQ is obtained by two factors (latent variables): the verbal comprehension index (VCI), whose observable variables are the results of the tests of vocabulary, similarities, information, and comprehension; and the working memory index (WMI), whose observable variables are the results for arithmetic, digit-span, and letter-number sequencing. PIQ is also obtained by two factors: the perceptual reasoning index (PRI), calculated over the results for picture completion, block design, matrix reasoning, visual puzzles, and figure weights; and the processing speed index (PSI), calculated over the results for coding, symbol search, and cancellation.

Nowadays, the Wechsler's tests validity is supported by the CHC theory (Weiss et al., 2006; Grégoire, 2013; Scheiber, 2016). The following relationship is suggested by Grégoire (2013): the verbal comprehension index (VCI) relates to crystallized intelligence (Gc); the working memory index (WMI) relates to short-term memory (Gsm); the perceptual reasoning index (PRI) relates to fluid intelligence (Gf) and visual processing (Gv); and the processing speed index (PSI) relates to processing speed (Gs). Scheiber (2016) suggests a slightly different relationship with the CHC model for the arithmetic subtest of the Wechsler Intelligence Scale for Children - 5th Edition (WISC-V), which would measure not only short-term memory (Gsm) but also fluid intelligence (Gf) and crystallized intelligence (Gc).

Incidentally, the study of psychic abnormalities and their correlation with neural anatomy—i.e., the anatomy-clinical method—has led to great findings from Charcot^1^ to modern computational neuroscience (Goetz, 2009). An example of the before-mentioned correlation between adjacent neural processes and intelligence tests is found in the following quote:

“The Picture Vocabulary subtest was developed to further reduce the nonspecific language demands of the Vocabulary test. (..) The child must have the capacity to interpret pictures into their semantic representation. (..) For the vocabulary subtests, there are a number of hypotheses to test when the examinee receives a low score (..): are low scores due to impaired auditory or semantic decoding, semantic productivity, access to semantic knowledge and/or executive functioning, or limited auditory working memory capacity?” (Weiss et al., 2006, p. 217).

The above description indicates how semantic processes relate to the sensory inputs of vision and hearing and executive, memory, and language functions. Therefore, the relevance of such diagnostic hypotheses goes beyond clinical assessment. It can inspire and direct research on the issues presented in Section 1, as the enhancement of computational models to explain semantic attribution and to enable semantic emulation in CI applications.

Finally, it is worth mentioning another point of convergence between the areas we are working with: the computer-assisted psychological assessment (CAPA), which includes not only the test administration but scoring, interpretation, and development. Such an approach can be exemplified by the recent works from Luo et al. (2020) and Martins and Baumard (2022). As pointed by Cohen et al. (2009, p. 78), “CAPA has become more the norm than the exception.” However, the authors identify some issues regarding this strategy that need to be addressed: information security concerns, comparability between the pencil-and-paper test and its computerized version; the validity of test interpretations by computational intelligence methods, and the unprofessional use of online psychological tests.

### 3.3. Intelligence measurement as the interface between humans and artificial systems

Two theoretical tests played an important role in AI development and speculated its limits: the Turing test and Searle's Chinese room. Initially published as “the game of imitation,” the Turing test proposes that a human observer interrogates a computer and another human without knowing their identities. Once the computer deceives the human observer, it could be claimed that it thinks or expresses human intelligent behavior (Turing, 1950).

Later, in the 1980s, when criticizing functionalism—a theory of mind that assumes that two isomorphic systems, given the same sensory inputs, would have identical mental states—John Searle defended the idea of biological naturalism—in which mental states are high-level abstractions that emerge from low-level instances, physically supported on neurons. For this, he proposed the Chinese room experiment, where a human knowing only the English language and equipped with an instruction book would perform operations written in Chinese. For an outside observer, the English-speaking human would be understanding Chinese and would pass the Turing test. However, the mere manipulation of symbols would not guarantee him any understanding (Russel and Norvig, 2009, p. 1031).

From a practical standpoint, when evaluating the interface between humans and artificial systems, rough comparisons are often used. For example, in formal reasoning tasks like arithmetic, human performance is compared to computational performance. In semantic-related applications, the algorithm's performance is typically measured against a dataset that one or more humans have annotated. These practical comparisons are in contrast to the philosophical debates about whether artificial intelligence can fully replace or emulate human intelligence, which often center around the Turing test and Searle's Chinese room experiment.

It sounds plausible to include in this section the answer given by an artificial intelligence agent itself, the ChatGPT^2^,^3^ (OpenAI, 2023) when asked about the differences between human and artificial intelligence:

(...) “Human intelligence is the product of the complex interactions between the brain's neurons and the environment, resulting in the ability to learn, reason, and solve problems. On the other hand, AI refers to the ability of machines or computer systems to perform tasks that typically require human-like intelligence, such as language processing, visual perception, decision-making, and problem-solving. (...) Finally, human intelligence is influenced by factors such as emotion, motivation, and context, whereas AI is typically designed to operate in a context-free environment and lacks emotional awareness or subjective experience.”

The above answer appears satisfactory and could easily be alleged to a student. In other words, it passes the Turing test, but as Searle advocated, it is nothing else than complex and effective symbol manipulation. From this perspective, a lack of a standard definition of intelligence between areas with distinct epistemic bases, as introduced in Section 1, sustains the problem posed by Searle. Computing seeks to generalize intelligence through its codification in algorithms. In formal reasoning (e.g., arithmetic), indeed, the result must be unanimous and general. However, on the other hand, evaluating the semantic performance of an algorithm trained from a dataset annotated by humans implies assuming that such annotations would be similar throughout humanity. It may be for some concepts, but numerous studies expose the subjective and particular character of any meaning attribution (Lakoff, 1990).

## 4. There is no consensus for intelligence

We presented and discussed the main theories for intelligence, summarized in Table 1 from computational intelligence and, in Table 2, from psychology. We propose a categorization of the intelligence scope based on the theory extension: general (G), open-domain (OD), or specific-domain (SD). Following this, we also categorized the modeling approach based on the epistemic foundations in the definition or theorization of intelligence, which can be either formal-logical (FL), instrumentalist (INS), information processing (IP), interactionist (INT), factor-analytical (FA), or skill-based (SB). For each author, we also identified the measurement approach, whether through psychometric (PSY), observational (OBS), or performance metrics (PM).

**Table 1:** Intelligence definitions from computational intelligence and their respective authors.

**References**	**Intelligence definition**	**Intel. scope**	**Mod. approach**	**Meas. approach**	**Related applications**
Bezdek (1992)	Distinguishes artificial, biological, and computational intelligences, according to their complexity, in a hierarchical structure.	G, OD, SD	IP	PM	Neural networks, pattern recognition, and general and specific-domain intelligences.
Russel and Norvig (2009)	The ability to perceive and act in a given environment through sensors and actuators, respectively, maximizing its expected utility.	OD, SD	FL	PM	Defines: simple reflex agents; model-based reflex agents; goal-based agents; and utility-based agents.
Chollet (2019)	Intelligence of a system is a measure of its skill-acquisition efficiency over a scope of tasks.	G	FL, IP, FA	PSY, PM	The author proposes the Abstraction and Reasoning Corpus (ARC) as a benchmark dataset for general intelligence. Uses the CHC model as a reference in his work.
Shneiderman (2020)	Categorizes AI into (i) emulation goal and (ii) application goal.	OP	FL	PM	For emulation: intelligent agent, humanoid robot, etc. For application: powerful tool, teleoperated device, etc.
Pandl et al. (2020)	Execution of tasks that are easy for people to perform but difficult to describe formally.	G, SD	FL, IP	PM	AGI (e.g., self-organizing and complex adaptive systems) and narrow AI (e.g., machine learning).

Intelligence scope can be general (G), open-domain (OD), or specific-domain (SD). The modeling approach can be information processing (IP), formal-logical (FL), factor-analytical (FA), interactionist (INT), instrumentalist (INS), or skill-based (SB). The measurement approach can be performance metrics (PM), psychometric (PSY), or observational (OBS). Related applications are detailed for each author.

**Table 2:** Intelligence definitions from psychology and their respective authors.

**References**	**Intelligence definition**	**Intel. scope**	**Mod. approach**	**Meas. approach**	**Related applications**
Binet and Simon (1904) apud (Cohen et al., 2009)	It is compounded by reasoning, judgment, memory, and abstraction.	G	INT	PSY	Has published the first IQ scale.
Boring (1961)	Criticized intelligence definitions: it is what tests test.	G	INS	PSY	Claimed for a better definition from the scientific community.
Spearman (1961)	There is a general factor *g* correlated with all its manifestations.	G	INS	PSY	Two-factor theory and *g* factor.
Wechsler (1958)	The aggregate or global capacity to act purposefully, think rationally, and deal effectively with the environment.	G, SD	INT, FA	PSY	Modeled the cognitive, executive, and conative aspects. Created the first editions of the WISC/WAIS psychometric tests.
Cattell (1941)	Intelligence has crystallized and fluid aspects.	OD	FA	PSY	The Gc-Gf model is part of the modern CHC model.
Piaget (2003)	It is developed through assimilation and accommodation processes.	OD, SD	INT	OBS	Psychology of intelligence through its development stages.
Horn (1965)	Added many specific factors to the Cattell's theory of Gc-Gf.	OD, SD	FA	PSY	This theory became known as Cattell-Horn model and did not admit the *g* factor.
Luria (1973)	Intelligence results from two ways of information processing: parallel or sequential.	OD, SD	IP	PSY, OBS	Luria is considered the precursor of neuropsychology.
Jensen (1978)	Intelligence is the first principal component of an indefinitely large number of highly diverse mental tasks.	G	FA	PSY	Distinguished intelligence from memory and learning. Also defined mental and physical abilities.
Howard (1983) apud (Jensen, 1978)	Defines multiple intelligences including art and spirituality.	OD	SB	OBS	There are several critics of his work due to the lack of psychometric validity.
Sternberg (2003, 2019)	It is compounded by analytic, creative, and practical aspects. Later, he defines intelligence through a biological and optimal perspective.	G, SD	IP	PSY, OBS	The triarchic theory of intelligence, in 2003, and the theory of adaptive intelligence, in 2019.
Carroll (2003)	Intelligence is divided into general, broad, and specific abilities.	G, OD, SD	FA	PSY	The three-stratum model is part of the modern CHC model.
Gottfredson (1997)	A highly general information processing capacity.	G, SD	FA, IP	PSY	Gottfredson advocates for the *g* factor.
Flynn (2007)	It integrates physiological, individual, and social aspects.	G, SD	IP	PSY	BIDS model and the Flynn effect.
McGrew (2009)	Unified the theories of Cattell-Horn and Carroll, creating the CHC model.	G, OD, SD	FA, IP	PSY, OBS, PM	This theory is the gold-standard reference for intelligence assessment in psychology.

Intelligence scope can be general (G), open-domain (OD), or specific-domain (SD). The modeling approach can be information processing (IP), formal-logical (FL), factor-analytical (FA), interactionist (INT), instrumentalist (INS), or skill-based (SB). The measurement approach can be performance metrics (PM), psychometric (PSY), or observational (OBS). Related applications are detailed for each author.

As a matter of fact, Tables 1, 2 lack a standard and general definition for intelligence, comprehensive to computational intelligence (mostly objective and formal) and psychology (mostly subjective). Since long ago, Neisser emphasized many times in his studies that a widely accepted definition of intelligence remains a challenge (Neisser, 1979; Neisser et al., 1996). In this paper, we reinforced such an idea by showing the many conceptualizations and divergences around this subject.

Flynn (2007, p. 50) observed that Jensen stopped using the term intelligence due to its lack of precision and consensus, referring to mental abilities the construct measured by *g*. The same absence is pointed out by Rindermann et al. (2020) in a survey from the Internet-based Expert Questionnaire on Cognitive Ability (EQCA), where different opinions on the *g* factor, intelligence measurement, and controversial issues were collected from the participants. Thus the models that define—and therefore explain—intelligence still need to be further elaborated. Other issues must also be taken, such as cross-cultural variation and human-machine interfaces. Flynn (2007, p. 54) says that “different societies have different values and attitudes that determine what cognitive problems are worth the investment of mental energy.” Following this, one can wonder if it is reasonable to think about a general intelligence.

Garlick (2002), after reviewing the main theories and models regarding intelligence, proposes a conceptual integration of the models from neural and cognitive sciences with the psychometric-based theory of general intelligence:

“These approaches initially seem contradictory because neuroscience and cognitive science argue that different intellectual abilities would be based on different neural circuits and that the brain would require environmental stimulation to develop these abilities. In contrast, intelligence research argues that there is a general factor of intelligence and that it is highly heritable. However, it was observed that if people differed in their ability to adapt their neural circuits to the environment, a general factor of intelligence would result. Such a model can also explain many other phenomena observed with intelligence that are currently unexplained” (Garlick, 2002, p. 131).

As the author emphasizes, further research is needed to provide an intelligence model that satisfactorily explains its manifestations. In neuropsychology, the CHC model has been considered the most robust and efficient one—we highlight here the role of psychological assessment and psychometrics to validate the theory a test is based upon since it brings empirical evidence. However, despite the broad and narrow abilities proposed by the CHC model, depicted in Figure 3, many of them are still unexplored by intelligence measurement instruments in psychology and even more rarely discussed in computational intelligence applications.

The naiveness of the concept of intelligence in computing is deeply debated in recent works (Korteling et al., 2021; Zhao et al., 2022). Its relationship with human intelligence is observed, for instance, from the following statement:

“In the early days of artificial intelligence, the field rapidly tackled and solved problems that are intellectually difficult for human beings but relatively straightforward for computers—problems that can be described by a list of formal, mathematical rules. The true challenge to artificial intelligence proved to be solving the tasks that are easy for people to perform but hard for people to describe formally—problems that we solve intuitively, that feel automatic, like recognizing spoken words or faces in images (Goodfellow et al., 2016, p. 1)”.

Since many AI and CI applications are related to cognitive and neuropsychic functions, such as vision, language, and decision-making, the equivalent robustness of the term intelligence found in psychology must be sought in computing for a consensus in the definition and measurement of intelligence among all its related areas.

## 5. Conclusions and future research

In this paper, we explored what intelligence is both in computational intelligence and psychology. Our first contribution was to show that, although these areas have a strong relationship, a common definition for intelligence is still needed. In the same way, we explored how intelligence is measured in these areas through instruments supported by the given theories. Secondly, we discussed that there is no consensus for intelligence even in the same area. This fact shows the need for a common concept to guide human and computational intelligence since divergent concepts about intelligence can bias measurements and mislead applications.

Based on this, we propose that intelligence is an agent's ability to process external and internal information to find an optimum adaptation (decision-making) to the environment according to its ontology and then decode this information as an output action. This definition is compatible with the CHC model. However, applying the CHC model in computational intelligence can be arduous, given that these agents have considerably different ontological and epistemological bases. Therefore, we propose a categorization of intelligence in the following aspects: (i) formal intelligence, based on reasoning and logical representation; (ii) semantic intelligence, characterized by meaning attribution for both vague and accurate information; (iii) contextual intelligence, an optimization scheme that takes into account an agent's ontology and the state of the environment; (iv) social or affective intelligence, which involves interaction between agents and is dependent on their respective ontologies; and (v) processing resources, the biological or digital substrates that enable any intelligent expression.

It is important to note that the definition we propose is the result of a theoretical synthesis of the studies presented in this paper while also considering the prevailing scientific paradigms and their inherent conflicts, such as objectivism vs. subjectivism, determinism vs. indeterminism, and formal-logics vs. information processing. We believe this categorization provides a comprehensive framework for further research on intelligence by promoting a collaborative perspective across different disciplines. In our future study, we aim to establish an empirical foundation for the proposed definition and encourage other researchers to explore this avenue as well.

Our proposal raises several research questions, including the possibility of a unified concept of intelligence that abstracts the ontology of each agent (i.e., the biological constitution vs. digital) and how to unify the measurements of this single theory. Additionally, we need to evaluate and differentiate narrow abilities from computational and human agents. Notably, the CHC model is a robust theory for intelligence, but many of its broad and narrow abilities are still underexplored in psychology and rarely mentioned in computational intelligence applications. These remaining questions must be addressed in future research.

## Author contributions

TD formulated the research manuscript idea, provided substantial edits to the manuscript and final draft, and aided in the interpretation of the manuscript. HL provided substantial edits to the manuscript and the final draft and aided in the interpretation of the manuscript. All authors approved the submitted version.

## Footnotes

^1^Jean-Martin Charcot (1825–1893) was a French neurologist who is considered a pioneer in the field of clinical neurology. He is known for his contributions to the understanding and treatment of several neurological disorders, including multiple sclerosis and Parkinson's disease. He was also a teacher and mentor to many influential figures in the field of medicine, including Sigmund Freud.

^2^ChatGPT is “a large-scale, multimodal model which can accept image and text inputs and produce text outputs,” OpenAI (2023) developed and trained under a deep learning perspective.

^3^The complete transcript of the interaction with the ChatGPT-3.5 prompt is available as Supplementary material for this paper.
