# Supplementary material for: Intelligence across humans and machines: a joint perspective
Source: Front Psychol. 2023 Aug 17;14:1209761. doi: 10.3389/fpsyg.2023.1209761 (PMC10470035; doi:10.3389/fpsyg.2023.1209761)
Supplement: Supplementary file 1 [file Presentation_1.pdf]

Supplementary Material

1 CHATGPT-3.5 PROMPT

This supplementary material contains the complete transcription of a conversation between a human user and the ChatGPT prompt. Each subsection is a question asked by the human user. The content of each subsection is the response given by the artificial agent. The conversation was recorded on the first author's OpenAI account, registered under the code *cd5546d7-1ed8-4bcf-b4b6-29922f7a4239*.

1.1 What is intelligence?

Intelligence is a multifaceted and complex construct that can be defined in different ways. At its core, intelligence refers to the ability to learn, understand, reason, and adapt to new situations and challenges. It involves a combination of cognitive abilities such as problem-solving, critical thinking, memory, language, and spatial skills, as well as social and emotional skills such as empathy, self-awareness, and emotional regulation.

There are many different theories of intelligence, including the well-known ”g factor” theory, which posits that intelligence can be measured by a single general factor, and the multiple intelligence theory, which suggests that intelligence can be divided into several distinct types, such as linguistic, logical-mathematical, musical, spatial, bodily-kinesthetic, interpersonal, and intrapersonal.

Intelligence is also influenced by a variety of factors, including genetics, environment, education, and culture. It is a dynamic and malleable trait that can be developed and improved through practice, learning, and experience.

1.2 What is the difference from human intelligence and artificial intelligence?

Human intelligence and artificial intelligence (AI) are fundamentally different in several ways. Human intelligence is the product of the complex interactions between the brain's neurons and the environment, resulting in the ability to learn, reason, and solve problems. On the other hand, AI refers to the ability of machines or computer systems to perform tasks that typically require human-like intelligence, such as language processing, visual perception, decision making, and problem-solving.

One of the main differences between human intelligence and AI is their underlying mechanisms. Human intelligence is based on the processing of information through neural networks in the brain, whereas AI relies on algorithms and computer programs that process data and perform operations based on pre-defined rules and decision trees.

Another key difference is that human intelligence is characterized by its flexibility and adaptability, allowing individuals to learn and adjust to new situations and challenges. In contrast, AI is generally designed to perform specific tasks or solve particular problems, and its abilities are limited to the scope of its programming.

Finally, human intelligence is influenced by factors such as emotion, motivation, and context, whereas AI is typically designed to operate in a context-free environment and lacks emotional awareness or subjective experience.

1.3 Would you be able to try a WAIS assessment?

As an AI language model, I am not able to administer a Wechsler Adult Intelligence Scale (WAIS) assessment, as this requires a trained professional with specific qualifications and expertise in psychological assessment. The WAIS is a standardized assessment tool that requires a specific set of instructions and guidelines to be followed to ensure its accuracy and reliability. It also requires a human administrator to interpret the results and provide appropriate feedback and recommendations.

However, I can provide you with general information about the WAIS, its components, and its use in psychological assessment. The WAIS is a widely used intelligence test designed to measure cognitive abilities in adults. It assesses several key areas of intelligence, including verbal comprehension, perceptual reasoning, working memory, and processing speed. The test consists of several subtests that measure specific abilities, such as vocabulary, arithmetic, block design, and digit span.

The results of the WAIS assessment can provide valuable information about an individual's cognitive strengths and weaknesses, as well as their overall intellectual functioning. However, it is important to note that the results of the WAIS should not be used in isolation to make important decisions about an individual's abilities, as other factors such as motivation, education, and experience can also influence performance on the test.

1.4 Could someone apply a WAIS or WISC assessment on you?

As an AI language model, I am not capable of being assessed using the Wechsler Adult Intelligence Scale (WAIS) or the Wechsler Intelligence Scale for Children (WISC). These tests are designed to measure human cognitive abilities and are administered by trained professionals to individuals who are capable of providing verbal and nonverbal responses to the test items.

As an artificial intelligence, I am not capable of experiencing emotions or responding to test items in the same way that a human would. My responses are generated based on statistical patterns in large datasets and are not reflective of a cognitive profile that could be assessed using standardized testing.

It is important to note that the use of psychological tests on artificial intelligence systems is not currently a recognized practice within the field of psychology, as these systems do not possess the same cognitive abilities or experiential factors as humans.

1.5 How could you categorize artificial intelligence or computational intelligence based on the CHC theory of intelligence?

The Cattell-Horn-Carroll (CHC) theory of intelligence is a comprehensive model of cognitive abilities that organizes them into three broad strata: narrow abilities, broad abilities, and a general factor (g). While this theory was originally developed to describe human intelligence, it has also been applied to the evaluation of artificial intelligence and computational intelligence.

Here are some examples of how different aspects of artificial intelligence or computational intelligence might be categorized based on the CHC theory of intelligence:

- Narrow Abilities: These are specific cognitive skills that are involved in particular tasks or domains. For example, in artificial intelligence, narrow abilities might include natural language processing, image recognition, speech synthesis, or game playing. These abilities might be further subcategorized based on their specific characteristics or demands.

- Broad Abilities: These are higher-order cognitive abilities that involve the integration and coordination of multiple narrow abilities. For example, in artificial intelligence, broad abilities might include problem solving, reasoning, decision-making, or learning. These abilities might be evaluated by assessing performance across multiple narrow abilities or tasks.

- General Factor (g): This is the overarching construct that reflects an individual's overall cognitive ability. In artificial intelligence or computational intelligence, this might be reflected in the ability of a system to perform well across a wide range of tasks or domains. However, it is important to note that the concept of g may not be directly applicable to artificial intelligence or computational intelligence, as these systems do not possess the same type of cognitive profile as humans.

Overall, the CHC theory of intelligence provides a useful framework for understanding the cognitive abilities that are involved in artificial intelligence and computational intelligence, and how these abilities can be evaluated and measured.

1.6 What is the best way to measure intelligence from machines and humans?

Measuring intelligence in both machines and humans can be challenging and involves different approaches and methodologies. Here are some commonly used methods for measuring intelligence in both machines and humans:

For humans: - Intelligence Tests: Standardized intelligence tests, such as the Wechsler Adult Intelligence Scale (WAIS) or the Stanford-Binet Intelligence Scale, are widely used to measure human intelligence. These tests assess a range of cognitive abilities, including verbal and nonverbal reasoning, problem-solving, and memory.

- Cognitive Tasks: These are specific tasks that are designed to measure different aspects of cognitive ability, such as working memory, attention, or decision-making. Cognitive tasks can range from simple tasks like digit span to more complex tasks like the Wisconsin Card Sorting Test.

- Behavioral Observations: Observing an individual's behavior in real-world situations can provide insight into their cognitive abilities. For example, monitoring a person's ability to navigate a complex environment or make decisions under time pressure can provide information about their cognitive abilities.

For machines: - Performance on Specific Tasks: In artificial intelligence, machine intelligence is often evaluated based on performance on specific tasks, such as natural language processing, image recognition, or game playing. The accuracy and speed with which a machine can complete these tasks can be used as a measure of its intelligence.

- Learning Curves: In machine learning, learning curves can be used to evaluate the performance of an AI system as it is trained on a particular task or dataset. A system that shows rapid improvement and reaches high levels of performance may be considered more intelligent than one that shows slower improvement or lower overall performance.

- Human Evaluation: In some cases, human evaluators may be used to assess the intelligence of a machine system. For example, in the Turing Test, human judges interact with a machine and a human through a text-based interface and attempt to determine which is which based on their responses.

It is important to note that intelligence is a complex and multi-faceted construct, and no single measure or method can fully capture all aspects of intelligence in either machines or humans. Multiple measures and methods are often used in combination to provide a more comprehensive understanding of intelligence in both domains.

1.7 Considering these differences between humans and machines, what are the common aspects of their intelligence? How to measure them?

While humans and machines have different types of intelligence, there are some common aspects of intelligence that can be measured in both. Here are some examples of these common aspects of intelligence and how they can be measured:

1. Problem Solving: Both humans and machines can demonstrate their intelligence through their ability to solve problems. In humans, problem-solving ability is often measured using standardized intelligence tests, such as the Wechsler Adult Intelligence Scale (WAIS). In machines, problem-solving ability can be measured by evaluating their performance on specific tasks or problems, such as solving puzzles or playing games.

2. Learning: Both humans and machines can learn from experience and adapt to new situations. In humans, learning ability is often assessed through cognitive tasks or by observing behavior in real-world situations. In machines, learning ability can be measured by evaluating their performance on specific tasks as they are trained on larger and more complex datasets.

3. Language and Communication: Both humans and machines can use language and communication to express ideas and interact with others. In humans, language ability is often measured using standardized language tests, such as the Peabody Picture Vocabulary Test (PPVT). In machines, language and communication ability can be measured by evaluating their performance on tasks such as natural language processing, speech recognition, and speech synthesis.

4. Perception and Recognition: Both humans and machines can perceive and recognize objects, patterns, and other stimuli in their environment. In humans, perception and recognition ability are often assessed using cognitive tasks or by observing behavior in real-world situations. In machines, perception and recognition ability can be measured by evaluating their performance on specific tasks, such as image or speech recognition.

5. Decision Making: Both humans and machines can make decisions based on available information and evaluate potential outcomes. In humans, decision-making ability is often measured using cognitive tasks or by observing behavior in real-world situations. In machines, decision-making ability can be measured by evaluating their performance on specific tasks, such as decision-making in game playing or problem solving.

Measuring these aspects of intelligence in both humans and machines often involves using different methods and techniques, depending on the specific task or domain. However, standardized tests, cognitive tasks, and real-world observations are commonly used to assess these common aspects of intelligence in both humans and machines.
